# Toward Generating More Diagnostic Features from Photoplethysmogram Waveforms

**DOI:** 10.3390/diseases6010020

**Published:** 2018-03-11

**Authors:** Mohamed Elgendi, Yongbo Liang, Rabab Ward

**Affiliations:** 1 Faculty of Medicine, University of British Columbia, Vancouver, BC V1Y 1T3, Canada; 2 BC Children’s & Women’s Hospital, Vancouver, BC V6H 3N1, Canada; 3 School of Electrical and Computer Engineering, University of British Columbia, Vancouver, BC V6T 1Z4, Canada; liangyongbo001@gmail.com (Y.L.); rababw@ece.ubc.ca (R.W.)

**Keywords:** photoplethysmography, pulse oximeter, clinical parameters, blood pressure estimation, global health, digital health, mobile health

## Abstract

Photoplethysmogram (PPG) signals collected using a pulse oximeter are increasingly being used for screening and diagnosis purposes. Because of the non-invasive, cost-effective, and easy-to-use nature of the pulse oximeter, clinicians and biomedical engineers are investigating how PPG signals can help in the management of many medical conditions, especially for global health application. The study of PPG signal analysis is relatively new compared to research in electrocardiogram signals, for instance; however, we anticipate that in the near future blood pressure, cardiac output, and other clinical parameters will be measured from wearable devices that collect PPG signals, based on the signal’s vast potential. This article attempts to organize and standardize the names of PPG waveforms to ensure consistent terminologies, thereby helping the rapid developments in this research area, decreasing the disconnect within and among different disciplines, and increasing the number of features generated from PPG waveforms.

## 1. Introduction

Photoplethysmography (PPG) waves contain a wealth of cardiovascular circulatory information, and many studies have shown its effectiveness in measuring and evaluating heart rate, blood oxygen saturation, blood pressure, cardiac output, arteriosclerosis, and vascular aging [1,2]. The PPG waveform itself is quite simple and not always informative, and therefore most clinicians check the derivatives of PPG waveforms to better evaluate the changes in the waveforms [3,4].

Applying the first derivative to the PPG waveform started in 1972 by Ozawa, and it was reported in Japanese [4]. The second derivative was then applied to the original PPG waveform to facilitate the interpretation and understanding of the original PPG waveform [4]. It is known that the main two waves of the PPG waveform are systolic and diastolic waves. Ozawa did not provide names for the first derivative waveforms. However, he suggested names for the second derivative of the PPG—namely *a* wave (early systolic positive wave), *b* wave (early systolic negative wave), *c* wave (late systolic reincreasing wave), *d* wave (late systolic redecreasing wave), and *e* wave (early diastolic positive wave).

Despite a marked increase of interest in this research field [5], there is a lack of uniformity in the terms used to refer to waveform types and their respective features. We therefore sought to investigate and survey the different terms used to refer to the PPG waveform and its derivatives in an attempt to create naming standards that can be used in the field.

Generally speaking, the acronym PPG is used to refer to the photoplethysmogram signal, as recommended in [6]. The first and second derivatives of the PPG signal are also studied, as they contain precise information about the characteristics within the PPG signal. For example, the PPG waveform indicates blood movement in the body’s vessels, whereas the first derivative of the PPG represents the velocity of blood flow in the finger. Thus, using the acronym VPG (velocity of PPG) to describe the first derivative of the PPG waveform is a fitting name choice. Likewise, it is fitting to use the acronym APG (acceleration plethysmogram) to refer to the second derivative of the PPG signal, which provides the acceleration of blood flow in the fingers [7]. Note that the use of the acronym APG was recommended in [6].

## 2. PPG Characteristics Terms

**Figure 1:**
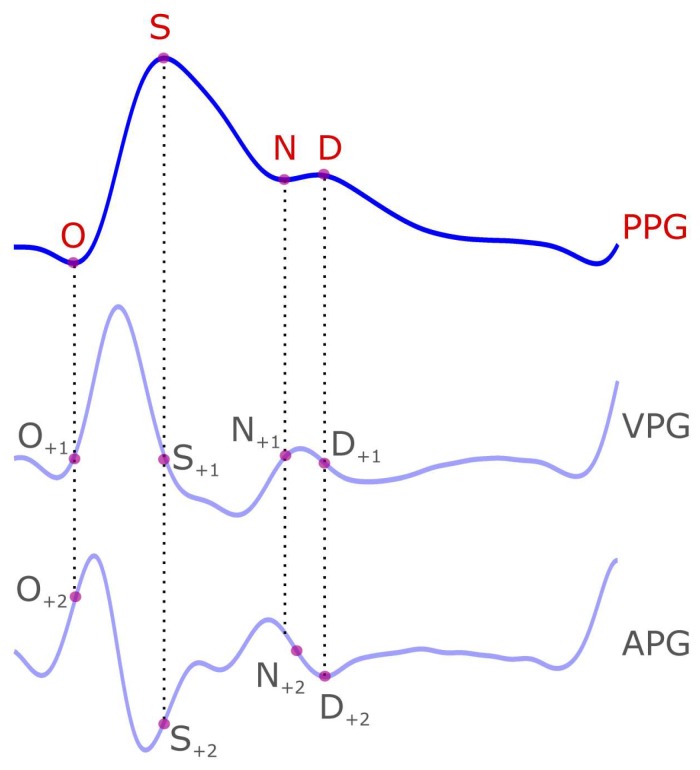
**Demonstration of the photoplethysmogram (PPG) signal and its four waveforms (O(nset), S(ystolic), N(otch), and D(iastolic)).** The four PPG waveforms are mapped on the velocity photoplethysmogram (VPG) and the acceleration photoplethysmogram (APG) signals. The subscript of +1 indicates the location of the PPG waveform on the first derivative of the PPG signal (i.e., VPG) while the +2 subscript indicates the location of the PPG waveform on the second derivative of the VPG signal (i.e., APG).

Many researchers focus on the morphological characteristics of the PPG to evaluate the cardiovascular function of the body [1,2,8,9,10]. One heartbeat PPG waveform consists of the onset of the systolic wave, systolic peak, dicrotic notch, and a diastolic peak [8]. Inconsistent naming is found with these PPG waveforms, and at time each wave is referred to by its description (or its function) as shown in Table 1. We therefore suggest the following institutive names to represent the characteristics respectively: O, S, N, and D. Here, O refers to Onset wave, S refers to Systolic wave, N refers to Notch, and D refers to Diastolic wave, as shown in Figure 1. Note that we use capital non-italic letters for the PPG waveform acronyms.

## 3. VPG Characteristic Terms

**Figure 2:**
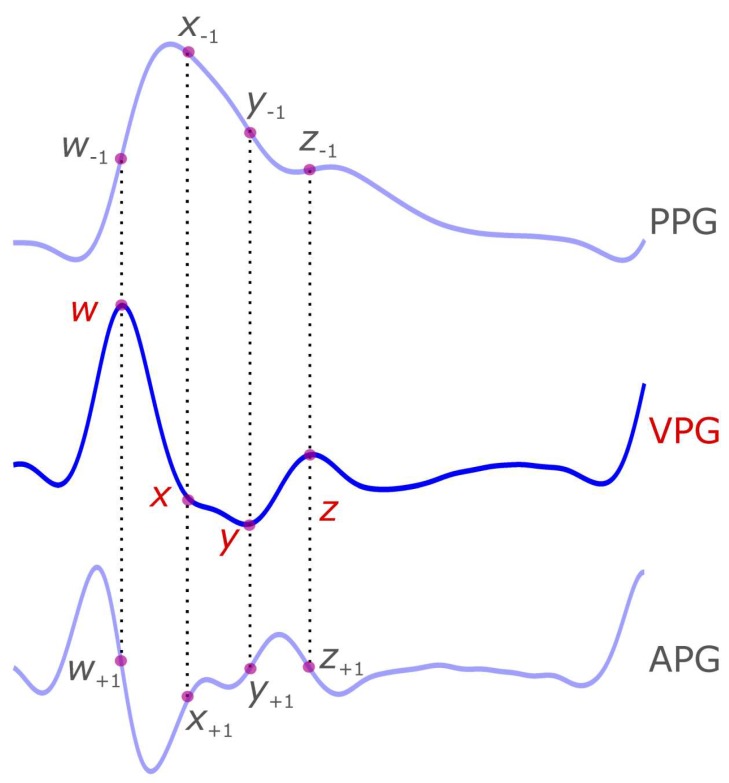
**Demonstration of the velocity photoplethysmogram (VPG) signal and its four waveforms (*w*, *x*, *y*, and *z*).** The four VPG waveforms are mapped on the photoplethysmogram (PPG) and the acceleration photoplethysmogram (APG) signals. The subscript of −1 indicates the location of the VPG waveform on the first integral of the VPG signal (i.e., PPG) while the +1 subscript indicates the location of the VPG waveform on the first derivative of VPG signal (i.e., APG). Note that the negative sign refers to the mathematical integration while the positive sign refers to the mathematical derivation.

The VPG waveform has been found to have more valuable information in recent research, and has several components that have not yet been officially named [11,12,13]. Based on our knowledge, there is no commonly used term to refer to the VPG components, perhaps limiting investigation on this topic. It is therefore necessary to refer to specific terminology when discussing VPG components (cf. Table 1). We suggest the use of *w*, *x*, *y*, and *z* (as shown in Figure 2) to refer to the maximum slope peak in systolic of VPG waveform, the local minima slope in systolic of VPG waveform, the global minima slope in systolic of VPG waveform, and the maximum slope peak in diastolic of VPG waveform, respectively. Note that we use small italic letters for the VPG waveform components because each is the result of applying a derivative.

## 4. APG Characteristic Terms

**Figure 3:**
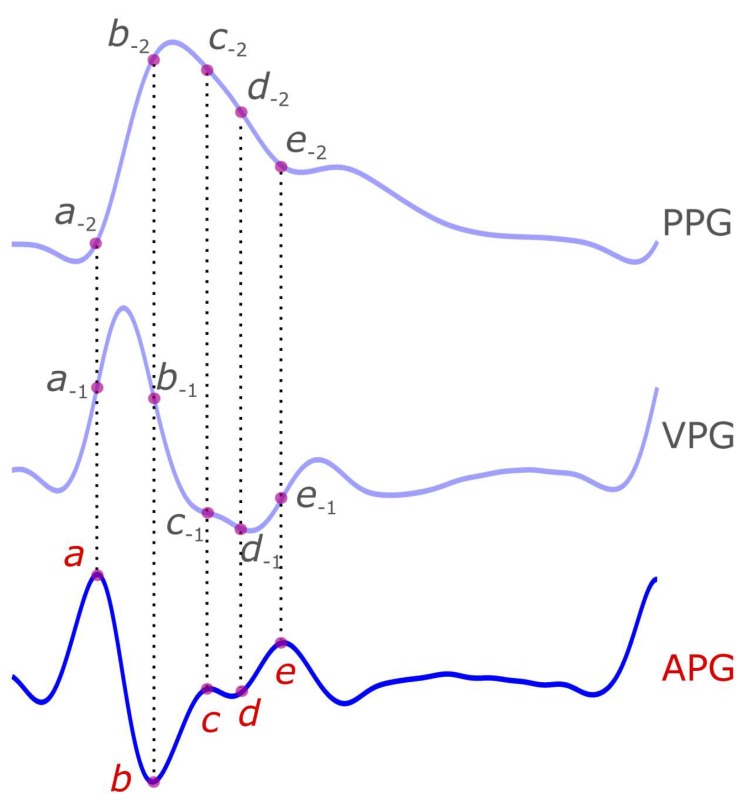
**Demonstration of the acceleration photoplethysmogram (APG) signal and its five waveforms (*a*, *b*, *c*, *d,* and *e*).** The five APG waveforms are mapped on the photoplethysmogram (PPG) and the velocity photoplethysmogram (VPG) signals. The subscript of −1 indicates the location of the VPG waveform on the first integral of the APG signal (i.e., VPG), while the −2 indicates the location of the APG waveform on the second integral of the APG signal (i.e., PPG). Note that the negative sign refers to the mathematical integration.

The waveforms of APG signal are well-established in the literature [6,8,14,15,16,17,18]. Every heartbeat in the APG signal consists of five waveforms: *a*, *b*, *c*, *d*, and *e* waveforms, as shown in Figure 3. The first waveforms (*a*, *b*, *c*, and *d*) represent a complete cycle of systole, while the *e* wave represents the onset of the diastole. Given there are already commonly used terms to refer to these waveforms, there is no need to rename or suggest other names/terminologies. Note that we use small italic letters for the APG waveforms because each is the result of applying a derivative.

## 5. Discussion

Screening through the literature for PPG features used in research, we can find two main approaches: *blind* and *insightful*. To explain the difference, we will discuss these approaches as follows:

### 5.1. Blind Approach

To our knowledge, the first work that applied the *blind* approach to PPG signals was introduced by Homma et al. [19], and researchers then followed this as reported in [20,21]. They developed a diagnostic system by feeding the APG signal to a neural network without caring about extracting or localizing features, which is the main aim of our research paper. Clinicians are interested to know about the specific features that correlate with the investigated diseases. By knowing the feature location, clinicians can improve the understanding of disease etiology and development, which consequently may open other areas of investigation, as opposed to investigations that do not focus on the importance of features.

### 5.2. Insightful Approach

To our knowledge, the first work that introduced the *insightful* approach was carried out by Ozawa et al. [4]. Later on, researchers looked at APG waveforms and started to examine the *b*/*a* ratio as an indicator for arterial stiffness [4,22], blood lead concentration [23], essential hypertension [20], and age progression [24]. Other scientists [4,24] found that the (*b*-*c*-*d*-*e*)/*a* index was useful for evaluating vascular aging and for screening of arteriosclerotic disease. More use of *insightful* features in clinical application can be found in [8].

We specifically discuss in this paper how to increase the number of features in disease screening and diagnosis, which may be of particular interest for scientists who are interested in this type of *insightful* investigation. Moreover, we are providing a naming convention for the proposed new features for four reasons:

(1) To reduce the effort needed to read and understand research outcomes from different fields.
(2) To enable scientists to focus more on discussing findings and not to worry about discussing the linguistics behind feature extraction.
(3) To enable a universal understanding of outcomes and findings so that they can be easily replicated, evaluated, and validated.
(4) To avoid future differences in feature naming.

For scientists who are interested in the *insightful* approach, naming the PPG waveform and its derivatives is an important first step to analyzing PPG signals. Researchers need to be consistent with the terms used to describe PPG features extraction, and be more precise with terms used during their analysis. This consistency is especially important to facilitate knowledge sharing in the field of PPG signal analysis. The potential with using standard naming for PPG waveforms for diagnosis is vast. It can be expected that such a standard naming scheme will lead to the discovery of unchartered features. In the realm of healthcare, extracting more features can lead to more efficient and accurate diagnosis and screening methods for cardiovascular diseases.

Nine years ago, Lu et al. published a paper on the possibility of calculating heart rate variability (HRV) using PPG signals when compared to ECG [12]. During this study, Lu et al. detected the systolic peak and used it as the heartbeat reference point, and subsequently investigated HRV using the systolic–systolic intervals as R-R intervals in ECG. However, their work only investigated one waveform, which we named S, as discussed above. With the publication of the proposed PPG waveforms naming scheme, researchers will be encouraged to investigate other waveform features that were not considered before. For example, researchers can investigate the slope of *de* within the APG signals but also the slope *de* in the VPG signal, which is *d_−_*_1_*e_−_*_1_, and/or the slope *de* in the PPG signal, which is *d_−_*_2_*e_−_*_2_. As can be seen, the number of features that can be extracted are increased dramatically, and this can lead to more diagnostic features and better understanding of the investigated disease.

After discussing the *blind* and *insightful* approaches, it is worth noting that there are scientists who have reported results without mentioning which part of the PPG signal (*blind* approach) or what PPG features (*insightful* approach) were used in their system [25]. The reasoning behind this is unclear. Computer scientists or engineers may not put much weight on the value of fully explaining and understanding the features used for detecting specific disease. However, as mentioned above, clinicians certainly need to understand what features are correlated with disease investigation.

With reference to the results published by Elgendi in [8], the findings were intended to serve as an aid for researchers for the development of their own systems. Some of this work can be seen in [26,27,28,29], where algorithms were created in the field of pulse oximeter and PPG signals processing. We expect that with this paper, the features proposed will even more so boost the development of algorithms that are understood by clinicians, computer scientists, and engineers. Each feature has unique meaning for both the systole and diastole components of the PPG signal, thus providing more insight and clinical validation for the development of algorithms or diagnostic systems. Note that we recently published a PPG database that is publically available for testing these features and for validation [30].

## 6. Conclusions

At present, there is inconsistent naming used to refer to the PPG waveform, its derivatives, and its characteristics. This inconsistent naming can create confusion and disconnect between researchers in the field, in addition to making it difficult to effectively compare results. We offer a solution to create more features from PPG waveforms that could help create more diagnostic indexes for the investigated disease. We proposed the most fitting and intuitive names, and consequently increased the number of features that could be generated from the PPG waveform. The finding of this article can increase the benefits and potential of using PPG signals for investigating different diseases.

## Figures and Tables

**Table 1:** Suggested wave names in photoplethysmogram signal and its derivatives.

Signal	Wave Name	Suggested Name
Photoplethysmogram (PPG)	systolic notch	O
	systolic main peak	S
	diastolic notch or dicrotic notch	N
	dicrotic peak or diastolic peak	D
First Derivative Photoplethysmogram (VPG)	Max slope point in systole	*u*
	Min slope point in diastolic	*v*
	Max slope in diastole or MDS	*w*
Second Derivative Photoplethysmogram (APG)	*a* wave	*a*
	*b* wave	*b*
	*c* wave	*c*
	*d* wave	*d*
	*e* wave	*e*
